# HER2 Expression in Squamous Cell Carcinoma of the Vulva: A Systematic Review and Meta-Analysis

**DOI:** 10.3390/cancers18132162

**Published:** 2026-07-06

**Authors:** Natalia Luisy Farias Müller, Maitha Al Sibani, Yousef Ayoub, Mariam Ayoub, Abdul Kareem Pullattayil, Farideh Tavangar, Anna Plotkin, Sophia George, Katarzyna J. Jerzak, Helen Mackay, Rania Chehade

**Affiliations:** 1 School of Medicine, University of Southern Santa Catarina (UNISUL), Palhoca 88137-270, Santa Catarina, Brazil; 2 Department of Medical Oncology, Queen’s University, Kingston, ON K7L 2V7, Canada; 3 Faculty of Life Sciences, University of Toronto Mississauga, Mississauga, ON L5L 1C6, Canada; 4 Health Sciences Library, Queen’s University, Kingston, ON K7M 5R7, Canada; 5 Lawrence Bloomberg Faculty of Nursing, University of Toronto, Toronto, ON M5T 1P8, Canada; 6 Department of Laboratory Medicine & Pathobiology, University of Toronto, Toronto, ON M5S 1A8, Canada; 7 Lakeridge Health, Oshawa, ON L1G 2B9, Canada; 8 Division of Gynecology Oncology, Department of Obstetrics, Gynecology and Reproductive Sciences, Miller School of Medicine, University of Miami, Miami, FL 33146, USA; 9 Sylvester Comprehensive Cancer Center, Miami, FL 33136, USA; 10 Department of Medical Oncology, University of Toronto, Sunnybrook Health Sciences Centre, Toronto, ON M4N 3M5, Canada

**Keywords:** squamous cell carcinoma of the vulva, HER2, biomarker, gynecological cancer

## Abstract

**Background:** Vulvar cancer is a rare gynecologic malignancy comprising 1–3% of all cases. No established standard exists for advanced disease, and treatment is often extrapolated from cervical cancer. Although HER2 overexpression is well defined in breast cancer and recognized across multiple solid tumors, its prevalence and significance in vulvar cancer remain unclear. Recent activity of HER2-directed antibody–drug conjugate Trastuzumab deruxtecan in solid tumors with an objective response rate (ORR) of around 37% highlights the need to better characterize HER2 expression in vulvar cancer. **Methods:** We performed a systematic search of Medline, Embase, and the Cochrane Library up to May 2025. Eligible studies included ≥10 vulvar cancer cases, predominantly vulvar squamous cell carcinoma (VSCC), excluding vulvar Paget’s disease, with available HER2 assessment by immunohistochemistry and/or in situ hybridization. Two reviewers independently screened the studies. A random-effects model was used to estimate pooled HER2 positivity. Heterogeneity was assessed using Cochrane’s Q and Higgins’s I^2^. **Results**: Of 506 records, nine retrospective studies including 769 patients with predominantly squamous cell carcinoma histology (98%, n = 752) met inclusion criteria. A total of 50 HER2-positive cases were observed. Median age at diagnosis of vulvar cancer was between 55 and 78, reported in three studies. Molecular profiling was limited. Among three studies with known TP53 status (n = 206), 59% of the tumors expressed TP53 (n = 122), and among two studies with known human papilloma virus (HPV) status (n = 128), 21% (n = 27) were HPV-positive. Six studies used American Society of Clinical Oncology/College of American Pathologists (ASCO/CAP) HER2 testing guidelines in breast cancer. Pooled HER2-positive expression across ASCO/CAP-based studies was 2% (95% CI: 1%, 3%) and for non-ASCO/CAP-based studies was 21% (95% CI: 2%, 52%). Exploratory pooled estimated proportion of HER2-positive expression was 5% (95% CI: 0.4%, 14%). There was substantial heterogeneity across studies, I^2^ value of 91.1% [95% CI: 85.4%; 94.6%], and no significant publication bias was observed (Egger’s test p = 0.364). This study could not assess prognostic value of HER2 overexpression in VSCC. **Conclusions**: HER2 positivity in VSCC appears uncommon but it remains to be fully explored. Standardized assessment using contemporary ASCO/CAP breast, endometrial-specific and/or gastric criteria are needed to clarify the prevalence of HER2-positive versus HER2-low/ultralow disease to inform potential use of HER2-targeted therapy.

## Simple Summary

Vulvar cancer, most commonly, squamous cell carcinoma (VSCC) is a rare malignancy with limited treatment options in the advanced setting. More recently HER2 targeting antibody-drug conjugate (ADC), Trastuzumab deruxtecan has shown benefit in HER2-expressing gynecological cancers. Through systematic review and meta-analysis, data on HER2 overexpression was pooled from nine retrospective studies in five countries encompassing 769 patients with predominantly VSCC, and 50 HER2-positive cases were observed. Pooled HER2 overexpression across studies using ASCO/CAP guidelines (n = 6) was 2% (95% CI: 1%, 3%) whereas studies not based on ASCO/CAP guidelines (n = 3) demonstrated a pooled prevalence of 21% (95% CI: 2%, 52%). Exploratory pooled estimated proportion of HER2-positive expression was 5% (95% CI: 0.4%, 14%). HER2 positivity in VSCC appears low but remains to be fully explored to clarify the prevalence of HER2-positive versus HER2-low/ultralow disease to inform potential use of HER2-targeted therapy in this patient population. Ultimately, further work to define the expression of ADC-related antigens can help identify patients with VSCC who could benefit from targeted therapeutic approaches.

## 1. Introduction

Vulvar cancer is a rare malignancy accounting for approximately 1–3% of gynecological malignancies [1], and its incidence has been increasing worldwide [2]. Squamous cell carcinoma represents the predominant histologic subtype of vulvar cancer; however, other histologies are also reported. Classification of vulvar cancer based on human papilloma virus (HPV) and protein 53 (p53) status has identified three clinically distinct subtypes of vulvar squamous cell carcinomas (VSCCs) [3,4]. HPV-positive/p53 wild type tumors have the most favorable outcome compared to intermediate outcome for HPV-negative/p53 wild type VSCC and the poorest outcome for HPV-negative/p53 mutant VSCC [3]; yet treatment strategies remain uniform for all VSCCs. Primary treatment of localized vulvar cancer includes surgical management of the lesion and assessment of groin lymph nodes [5]. When complete surgical resection is not possible, treatment with combined chemotherapy and radiotherapy is considered [6]. Although prognosis is generally favorable for patients diagnosed with early-stage vulvar cancer, those presenting with recurrent or metastatic disease have short overall survival with limited availability of systemic therapy options [7]. Current treatments in the advanced setting are largely extrapolated from cervical cancer, highlighting the need for biomarker identification and development of novel targeted therapies for patients with advanced VSCC.

Various biomarkers are currently under investigation in squamous cell carcinoma [8,9]. Human epidermal growth factor receptor 2 (HER2) is a transmembrane receptor involved in cell growth, proliferation, differentiation and survival [10]. Historically, HER2 overexpression has been well characterized in breast carcinomas and is associated with aggressive behavior of cancer [10,11,12]; however, the emergence of HER2-targeted therapies has improved outcomes in this patient population [11]. Since then, HER2 overexpression and/or gene amplification has been identified in various solid tumors, including breast (15–20%) [13,14], bladder (6.7–37.5%) [15], lung (2.5–34%) [16], gastric/gastroesophageal junction (22%) [17], and uterine cancers (28%) [18]. HER2 overexpression rate tends to be higher in adenocarcinoma compared to squamous cell carcinoma histology; for example, HER2 overexpression was reported in 18% of lung squamous cell carcinoma compared with 34% of lung adenocarcinoma [19]. In cervical cancer, pooled HER2 overexpression was 4.1% in squamous cell carcinoma histology compared to 10.3% in non-squamous histology [20]. HER2-targeting agents such as monoclonal antibody Trastuzumab have improved outcomes for patients with HER2-positive cancers in breast [21], gastrointestinal (GI) [17,22] and uterine cancers [23]. Neratinib, an irreversible pan-HER tyrosine kinase inhibitor, has demonstrated durable response in HER2-mutated cervical cancer [24]. With recent identification of a promising response to HER2 antibody–drug conjugate (ADC) Trastuzumab deruxtecan (T-DXd) in various solid tumors, including gynecological cancers [25], comprehensive understanding of HER2 expression in VSCC has become an area of interest as it may guide the development of future targeted therapy.

The American Society of Clinical Oncology (ASCO) and the College of American pathologists (CAP) guidelines on HER2 testing in breast cancer were initially published in 2007, followed by updates in 2013, 2018, and 2023 to standardize identification of HER2 overexpressing tumors using a combination of tests involving immunohistochemistry (IHC) and in situ hybridization (ISH) [26,27,28,29]. Generally, HER2-positive expression is defined as IHC 3+ or IHC 2+ with ISH-positive status [28,30].

In accordance with the 2007ASCO/CAP guidelines on resection/excision specimens, HER2 testing by IHC was defined as follows: 0 = no staining; 1+ = weak, incomplete membrane staining in any proportion of tumor cells; 2+ = weak to moderately complete membrane staining in >10% of tumor cells; 3+ = strong, complete membrane staining in >30% of tumor cells [26]. The 2013 and subsequent ASCO/CAP updates were largely based on core biopsies as follows: 0 = no staining or incomplete and faint/barely perceptible membrane staining in ≤10% of tumor cells; 1+ = faint/barely perceptible incomplete membrane staining in >10% of tumor cells; 2+ = circumferential, incomplete and/or weak/moderate membrane staining in >10% of tumor cells or complete and circumferential intense membrane staining in ≤10% of tumor cells; 3+ = complete, intense staining of >10% of tumor cells [27,28].

For breast tumors with IHC 2+ staining, in situ hybridization (ISH) is used to determine HER2 amplification status. ISH-positivity is defined as average HER2 copy number ≥ 6.0 signals/cell or average HER2 copy number ≥ 4.0 signals/cell and HER2/chromosome 17 centromere (CEP17) ratio ≥ 2.0 [27,28]. The 2018 ASCO/CAP guideline further clarified interpretation of ISH equivocal or ISH complex/unusual pattern using concomitant IHC [28]. The 2023 guideline acknowledged a new indication of T-DXd following demonstration of improved survival in metastatic breast cancer with HER2 IHC 1+ or 2+ with ISH-negative status, known in DESTINY-Breast04 as HER2-low [14,29]. HER2-ultralow was defined in DESTNY-Breast06 [31] as IHC0 with membrane staining (IHC > 0 < 1+).

The spectrum of HER2-low/ultralow expression [29] in gynecologic malignancies, including vulvar cancer, remains to be fully explored. Furthermore, HER2-testing criteria have also evolved in gastric and endometrial cancers with scoring based on basolateral membranous staining, type of tissue (biopsy versus excisional) and cutoff threshold for HER2 positivity [30,32]. Clinically, 2007-modified ASCO/CAP-based criteria in the breast was used in the phase II randomized trial of Trastuzumab combined with doublet platinum-based chemotherapy and compared to chemotherapy alone in advanced uterine serous carcinoma [23], whereas the IHC part of 2016/2018 ASCO/CAP gastric criteria were used in the phase II DESTINY PanTumor02 of T-DXd [25]. There is ongoing discussion about which guidelines should be used across various gynecological cancers; there is no uniform guideline for HER2 testing in vulvar cancer. This may be further delineated with prospective biomarker-driven trials exploring the response rate to HER2-targeted therapies based on scoring criteria.

We hypothesize that biomarker-driven profiling of VSCC would improve access to targeted therapy. While HER2 overexpression has been characterized in vulvar Paget’s disease, with overexpression noted in 40% of invasive tumors [33], it remains to be explored in VSCC. The aim of this study was to determine the proportion of HER2 overexpression in a predominantly VSCC population through a systematic review and meta-analysis.

## 2. Methods

A literature search on Medline, Embase, and the Cochrane Library for all articles on HER2 expression in vulvar cancer from inception to May 2025 was conducted in addition to screening reference lists of included studies and relevant systematic reviews to identify additional studies. The completed search strategy is provided in (Supplementary Tables S1–S3). Studies were screened by two independent reviewers (Y.A. and M.A.). Inclusion criteria required studies with data available on HER2 expression by IHC and/or ISH. Studies were required to include ≥10 patients with VSCC and available HER2 expression data, excluding Paget’s disease of the vulva due to available literature review on the topic [33]. The Preferred Reporting Items for Systematic Reviews and Meta-Analysis (PRISMA) guidelines were followed for screening and inclusion of papers in the final analysis and synthesis [8] (Supplementary Tables S1–S4). The study was prospectively registered with PROSPERO (CRD420261391687).

### Statistical Analysis

The random-effects model was conducted to estimate the exploratory pooled proportion of HER2-positive expression in patients with VSCC. A subgroup analysis was performed to explore whether the proportion of HER2-positive VSCC differed according to ASCO/CAP guidelines in breast carcinoma status (Yes vs. No). Notably the first ASCO/CAP guideline on HER2 testing in breast cancer was in 2007, and HER2 assessment prior to that did not follow standardized protocols for scoring, tissue processing and quality control. Studies using methodologies prior to or different from ASCO/CAP-based guidelines were identified as non-ASCO/CAP-based in a subgroup analysis. Subgroup differences were evaluated using the Q-test and a random-effects model. The proportion of between-study heterogeneity was assessed using pseudo—*R*^2^. An exploratory meta regression analysis was conducted to assess whether differences in HER2 testing methodology contributed to the observed between-study variability. Heterogeneity was quantified using Cochrane’s Chi-squared test (Cochran’s Q) and Higgins’s I^2^ statistic. The fixed-effect model was used when heterogeneity was considered non-significant with a *p* > 0.05 combined with an I^2^ < 50 [34,35]. Forest plots were generated to depict study-specific proportions, overall pooled estimates and subgroup pooled estimates according to ASCO/CAP guideline status.

Risk of bias (RoB) was assessed by two authors (M.A. and R.C.). Retrospective studies were assessed using the Newcastle-Ottawa Scale based on several parameters including patient selection, comparability, and outcome/exposure [36]. Points were calculated for each study and classified as low, intermediate, high, or unable to score RoB accordingly. Disagreements were resolved through discussion until consensus between the authors was reached. Moreover, publication bias was explored using funnel plot asymmetry and Egger’s test. A *p* > 0.05 suggests no significant asymmetry and less evidence for publication bias [37]. All statistical analyses were performed in R (version 4.5.1). The “metaprop” and “metareg” functions from the “meta” package were applied to perform a meta-analysis of proportions using the Freeman–Tukey double arcsine transformation and meta regression analysis, respectively.

## 3. Results

**Figure 1:**
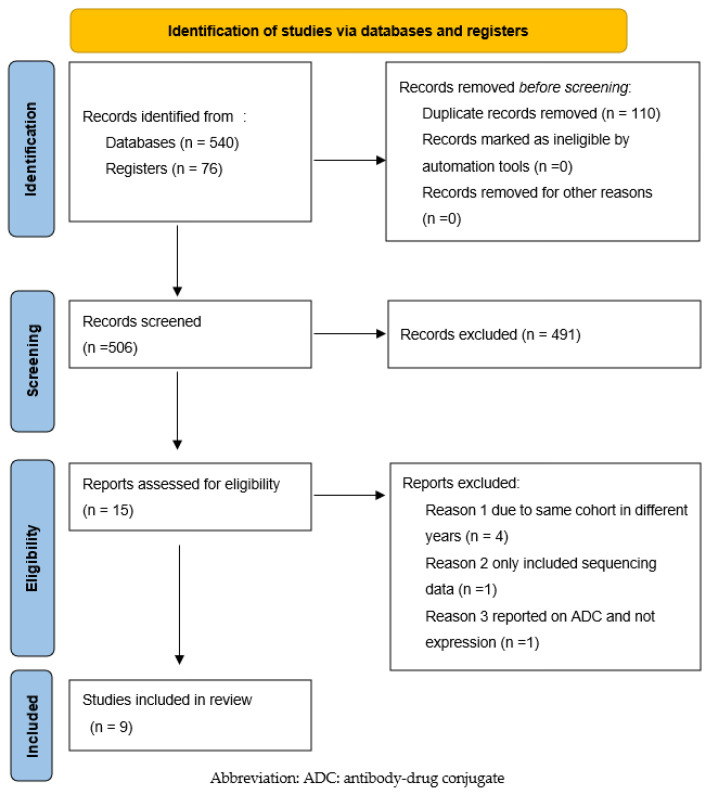
Prisma diagram of the study selection process.

Among 506 unique search results, 491 were excluded after title and abstract screening, and six were excluded after full text review (Figure 1). Nine retrospective studies met the inclusion criteria. To avoid overlapping populations between studies that included similar cohorts, only the most recent one was included in the meta-analysis for any two similar studies. In total, this meta-analysis included nine studies encompassing 769 patients treated across five countries with publication between 1990 and 2025 and 50 HER2-positive cases in total were observed (Table 1). Six studies included HER2-expression assessment based on ASCO/CAP guidelines in breast carcinoma; two studies used the 2007 guidelines where the HER2 positivity cutoff threshold required IHC expression of HER2 in >30% of cells [38,39] and three studies used more recent guidelines (2013 and subsequent) where the HER2 positivity cutoff threshold required HER2 IHC expression in >10% of cells [40,41,42]. One study did not specify the year of the ASCO/CAP guideline that was used to determine HER2 expression [43]. Three studies included HER2 expression assessment that was either prior to or not based on ASCO/CAP guidelines, including one study that used “light” or “heavy” as qualitative descriptions [44], while another study used HER2 membrane staining with ≥70% of cells as a cutoff for positive IHC expression [45] and the final study did not specify a cutoff [46].

Across nine studies of vulvar cancer, squamous cell carcinoma was the dominant histologic subtype (98%, n = 752), while adenocarcinomas represented only a small minority. Median age at diagnosis of vulvar cancer was between 55 and 78, as reported in three studies. Lymph node involvement was reported in six studies, including 361 patients where lymph node status was positive in 48% (n = 173). Molecular profiling was limited. Among three studies with known TP53 status (n = 206), 59% of vulvar cancers expressed TP53 (n = 122), and among two studies with known HPV status (n = 128), 21% (n = 27) were HPV-positive (Table 1 and Table S4).

**Figure 2:**
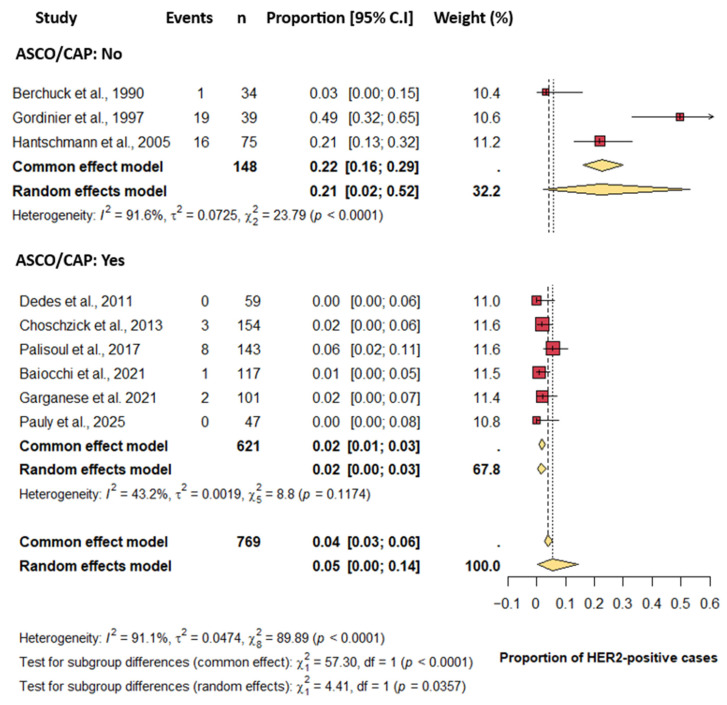
Prevalence of HER2 overexpression in vulvar squamous cell carcinoma across nine studies in the overall population and subgroup classification based on ASCO/CAP guidelines (No/Yes) with 95% confidence intervals. n, number of cases; C.I., confidence interval. References for studies from top to bottom [38,39,40,41,42,43,44,45,46]. Squares are the effect size of the individual studies; diamonds, the summarized effect size; horizontal lines, upper and lower borders of 95% CI; *p*-values < 0.05 are considered statistically significant. Abbreviation 95% C.I.: confidence interval, ASCO/CAP: ASCO/CAP: American Society of Clinical Oncology/College of American pathologists.

Meta-analysis results showed that the estimated pooled proportion of HER2 positivity for studies that used ASCO/CAP guidelines was 2% (95 CI: 1%, 3%) whereas studies that were not based on ASCO/CAP guidelines showed a substantially higher pooled estimate of 21% (95% CI: 2%, 52%); the exploratory overall pooled estimated proportion of HER2-positive status was 5% (95% CI: 0.4%, 14%). Excluding conference abstracts did not impact the estimated pooled proportion of HER2 positivity regardless of ASCO/CAP status. Figure 2 depicts the forest plot of the proportion of HER2-positive cases across nine studies along with two subgroups based on ASCO/CAP guideline status. Subgroup analysis demonstrated substantial differences in the pooled HER2-positive proportions according to ASCO/CAP status (Cochran’s Q-test: Q = 4.41, df = 1, *p* = 0.0357). Meta regression further confirmed that ASCO/CAP guideline status significantly contributed to between-study heterogeneity (*p* < 0.0001) and explained a major portion of heterogeneity (R^2^ = 68%). Overall, there was substantial heterogeneity across studies with an I^2^ value of 91.1% [95% CI: 85.4%; 94.6%] (Q = 89.9, df = 8, *p* < 0.0001).

**Figure 3:**
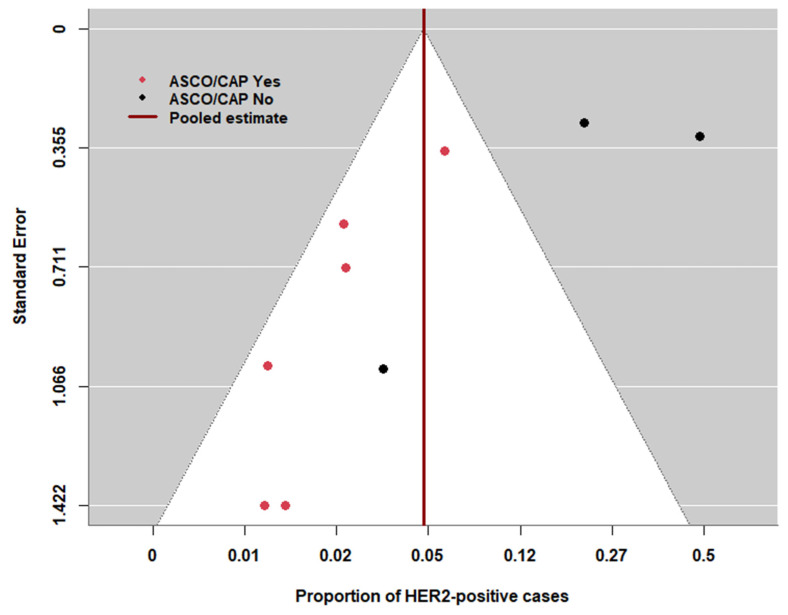
Funnel plot of proportion of HER2-positive cases among cohort studies as stratified by ASCO/CAP status. Abbreviation ASCO/CAP: ASCO/CAP: American Society of Clinical Oncology/College of American pathologists.

Egger’s regression test did not demonstrate statistically significant publication bias (Egger’s test *p* = 0.364). Figure 3 shows a funnel plot with asymmetry likely driven by two studies with substantially higher proportions of HER2-positive cases.

Across the included studies, RoB levels varied according to study design (Supplementary Table S5). Gordinier et al. [45], Palisoul et al. [40], Garganese et al. [42] and Baiocchi et al. [39] had low risk of bias whereas Berchuck et al. [44], Choschzick et al. [38] and Hantschmann et al. [46] had moderate risk of bias. Retrospective study design, small sample size, limited clinical outcome correlation and lack of long-term follow-up were contributing factors to bias assessment. Dedes et al. [38] and Pauly et al. [41] were abstract-based and could not be scored.

This study could not assess the prognostic value of HER2 overexpression in VSCC. In two studies that reported on outcome [39,46], the extent of HER2 expression in VSCC was not associated with overall survival. Two studies including 73 patients reported on the association of HER2 positivity with nodal and distant metastases [38,44]. One study that included 143 patients with vulvar cancer reported an association between HER2-positive status with adenocarcinoma histology [40].

## 4. Discussion

**Figure 4:**
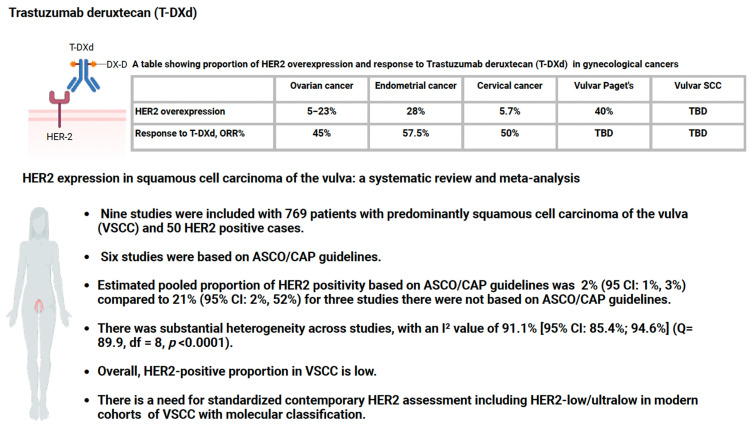
A schematic summary of the study. The table in the figure shows the proportion of HER2 overexpression across gynecological cancers and corresponding response to Trastuzumab deruxtecan. TBD refers to unknown status that remains to be determined. References for HER2 expression in ovarian, endometrial, cervical and invasive vulvar Paget’s disease, as well as reference for corresponding response to Trastuzumab deruxtecan are as follows: [18,20,25,33,47]. Abbreviation: T-DXd: Trastuzumab deruxtecan, HER2: human epidermal growth factor receptor 2. SCC: squamous cell carcinoma, ORR: overall response rate, VSCC: vulvar squamous cell carcinoma, ASCO/CAP: American Society of Clinical Oncology/College of American pathologists.

In our analysis (Figure 4), the estimated pooled proportion of HER2 positivity in studies employing ASCO/CAP guidelines was 2% (95% CI: 1%, 3%), whereas non-ASCO/CAP-based studies reported a much higher pooled rate of 21% (95% CI: 2%, 52%). Across all the studies, the exploratory pooled estimated proportion of HER2 positivity across all studies was 5% (95% CI: 0.4%, 14%). This prevalence is lower than that observed in other gynecological cancers, such as cervical (5.7%) [20], uterine (28%) [18], ovarian cancer (5–25%) [47] and invasive vulvar Paget’s disease (30–40%) [33]. Given the substantial methodological heterogeneity across studies, specifically with respect to ASCO/CAP status, and the small number of studies, the overall pooled HER2-positive estimate (5%) must be interpreted with caution and remains strictly exploratory. The observed significant differences based on ASCO/CAP status indicate that methodological variability may substantially influence pooled proportion estimates.

Early investigations conducted before the standardization of ASCO/CAP criteria often reported a trend toward higher HER2 positivity. For example, Berchuck et al. (1990) used “light” versus “heavy” staining criteria and described a metastatic VSCC case with strong HER2 expression (n = 1/34) [44], while Gordinier et al. (1997) used HER2 membranous staining with a cutoff ≥70% of cells for cases to be considered positive, reporting HER2 positivity in nearly half of VSCCs, noting an association with lymph node metastasis [45]. Hantschmann reported on cytoplasmic HER2 staining in 21.3% of the cases [46]. Data regarding key variables, including HPV status and p53 alterations, were limited.

More contemporary cohorts using ASCO/CAP-based evaluation, which incorporated standardized scoring of HER2 IHC with or without confirmation of gene amplification by ISH, demonstrated lower rates of HER2 positivity. Baiocchi et al. (2021) [39] and Choschzick et al. (2013) [38] reported HER2 IHC 3+ and/or amplified rates of 0.9% and 1.9%, respectively, using a >30% cutoff threshold for positivity with strong complete, intense membrane staining. On the other hand, Dedes et al. (2011) [43], Palisoul et al. (2017) [40], and Garganese et al. (2021) [42] reported HER2 IHC 3+ and/or amplified rates as 0%, 5.6%, 2%, respectively, using a >10% cutoff threshold for positivity with strong membrane staining for HER2 for cases to be considered positive. Palisoul et al. [40] included vulvar adenocarcinoma histology and metastatic cases which may account for a higher proportion of HER2 positivity. In the most recent analysis, Pauly et al. (2025) [41] did not identify any HER2-positive VSCC cases (among 47 cases examined) based on the ASCO/CAP 2016/2018 guidelines in breast carcinoma, but most cases were lymph node-negative, suggesting the need to delineate expression in early versus advanced stages, in addition to molecular classification by HPV and TP53 status.

In uterine cancer, HER2 positivity is associated with *TP53* mutations [18]; whether a similar differential expression exists across molecular subtypes of VSCC remains to be fully characterized. Definitively evaluating the prognostic value of HER2 in this disease will require large-scale, multi-center collaborations.

While next-generation sequencing (NGS) data were not aggregated in this study, there are genomic reports of *ERBB2* (Erb-B2 Receptor Tyrosine Kinase 2, commonly known as HER2) alterations in vulvar cancer. Ordi et al. reported a HER2 amplification rate of around 11.6% (n = 7/105) [48]. In another study of 82 cases of recurrent cervical and vulvovaginal cancers, including nine vulvovaginal cases, alterations in *PIK3CA* (phosphatidylinositol-4,5-bisphosphate 3-kinase catalytic subunit alpha), *ERBB2*, *AKT* (AKT Serine/Threonine Kinase), and *FGFR3* (Fibroblast Growth Factor Receptor 3) via NGS were detected in 35% (n = 29) [49] of cases. Identifying an NGS panel for potential new targeted therapies in VSCC remains to be fully explored.

Ultimately, VSCC is a rare type of gynecological malignancy with limited systemic therapy options in the advanced setting. Although standardized criteria for evaluating HER2 by IHC in VSCC have yet to be formally established, our study demonstrates a low HER2-overexpression rate. Moreover, because these studies were conducted before widespread recognition of HER2-low/ultralow disease, this study did not have granular data to assess these entities in VSCC. There are case reports on the response to T-Dxd in mammary-like vulvar cancer [50,51]. With the emerging data on HER2-low and HER2-ultralow expression in the context of response to T-Dxd, exploring the proportion of HER2-low/HER2-ultralow expression in VSCC cases is warranted for future biomarker-driven research. Beyond HER2, there are several alternative surface antigen targets in gynecologic cancers including B7 Homolog 4 (B7H4), folate receptor (FOLR1), trophoblast cell-surface antigen 2 (TROP2), tissue factor (TF), nectin-4 and epidermal growth factor receptor (EGFR) [6,52,53,54,55]. Historically, patients with vulvar cancers have not been included in clinical trials. Recent ADC-based trials such as DESTINY Pan-Tumor02 included vulvar cancer [25], and there is a prospective trial of EGFR targeting ADC Becotatug Vedotin combined with immunotherapy Zimberelimab in the treatment of recurrent and metastatic cervical cancer, vulvar cancer and vaginal cancer [56,57]. A better definition and understanding of surface antigen biomarker distribution in vulvar cancers and inclusion of vulvar cancers in multi-tumor clinical trials are essential if we are going to develop new therapies for this disease.

The primary strength of this study includes a rigorous systematic review focused on a rare under-studied patient population. Conversely, several limitations include the retrospective nature of the studies, small study size to perform meta regression analysis, potential publication bias risk including two studies relying on conference abstract data, the small number of HER2-positive events leading to very wide confidence intervals that limit precision, in addition to limited reporting on HPV and TP53 status. Notably, studies included various methods for HER2 IHC testing and scoring, spanning time before and after ASCO/CAP guidelines for HER2 testing in breast cancer were established.

Overall, the low rate of HER2 overexpression in VSCC (2%) reveals the need for standardized biomarker assessment in modern cohorts with uniform scoring criteria and molecular classification. Given low prevalence, this would be challenging for biomarker-driven clinical trials requiring large sample sizes; rather, prospective trials would focus on identifying rare molecularly selected subgroups that may be relevant for basket trials.

## 5. Conclusions

HER2 overexpression is low at 2% in VSCC, as determined through a systematic review and meta-analysis of six studies based on ASCO/CAP guidelines, with limitations of a small sample size, risk of bias and a lack of a uniform scoring criterion. This value is lower than that of invasive vulvar Paget’s, where HER2 overexpression is around 40%. Moving forward, there is a need for standardized HER2-testing protocols in vulvar cancer. Evaluating HER2-low/ultralow expressions in a modern cohort with available molecular classification may inform responses to HER2-targeting ADCs in a rare cancer. Ultimately, further work is needed to define the expression of ADC-related surface antigens; this can help identify patients with VSCC who could benefit from targeted therapeutic approaches.

## Figures and Tables

**Table 1:** A table of studies that reported HER2 expression among patients with predominantly vulvar squamous cell carcinoma.

Study by Author	Country	Year	Total n	ASCO/CAP Guidelines (Yes/No)	Cutoff Threshold for HER2 Positivity	HER2-Positive (n)	% HER2-Positive
Berchuck et al. [44]	USA	1990	34	No	Heavy staining.	1	2.9%
Gordinier et al. [45]	USA	1997	39	No	Membrane staining ≥70% of cells.	19	48.7%
Hantschmann et al. [46]	Germany	2005	75	No	Cutoff not mentioned.	16	21.3%
Dedes et al. [43]	Switzerland	2011	59	Yes	Cutoff not mentioned.	0	0%
Choschzick et al. [38]	Germany	2013	154	Yes	Strong complete membranous staining in more than 30% of cells.	3	1.9%
Palisoul et al. [40]	USA	2017	143	Yes	Strong complete membranous staining in more than 10% of cells.	8	5.6%
Baiocchi et al. [39]	Brazil	2021	117	Yes	Strong complete membranous staining in more than 30% of cells.	1	0.9%
Garganese et al. [42]	Italy	2021	101	Yes	Strong complete membranous staining in more than 10% of cells.	2	2%
Pauly et al. [41]	Germany	2025	47	Yes	Strong complete membranous staining in more than 10% of cells.	0	0%

Abbreviations: n, number of cases; ASCO/CAP, American Society of Clinical Oncology/College of American Pathologists (ASCO/CAP); HER2, human epidermal growth factor receptor 2; % HER2-positive, percentage of HER2-positive cases.

## Data Availability

Data is contained within the article.

## Supplementary Materials

The following supporting information can be downloaded at: https://www.mdpi.com/article/10.3390/cancers18132162/s1, Table S1: PRISMA 2020 for Abstracts Checklist; Table S2: PRISMA 2020 Checklist; Table S3: Search strategy, Table S4: A table of studies that reported on HER2 expression among patients with predominantly vulvar squamous cell carcinoma with associated clinicopathological characteristics; Table S5: Newcastle-Ottawa Scale assessment of cohort studies.
