# Supplementary material for: HER2 Expression in Squamous Cell Carcinoma of the Vulva: A Systematic Review and Meta-Analysis
Source: Cancers (Basel). 2026 Jul 6;18(13):2162. doi: 10.3390/cancers18132162 (PMC13359448; doi:10.3390/cancers18132162)
Supplement: Supplementary file 1 [file cancers-18-02162-s001.zip › cancers-4271133-Supplementary Material.pdf]

Supplementary Material

HER2 Expression in Squamous Cell Carcinoma of the Vulva: A Systematic Review and Meta-Analysis

Summary

**Table S1**: Abstract Prisma Checklist	Page 2
**Table S2:** Preferred Reporting Items for Systematic Reviews and Meta-Analysis (PRISMA) Checklist for the Manuscript	Pages 3, 4, 5
**Table S3**: Full Search Strategy	Page 6, 7
**Table S4**: A table of studies that reported on HER2 expression among patients with predominantly vulvar squamous cell carcinoma with associated clinicopathological characteristics.	Page 7
**Table S5**: Newcastle-Ottawa Scale assessment of cohort studies	Page 7, 8

Table S1: Abstract Prisma Checklist

Section and Topic	Item #	Checklist item	Reported (Yes/No)
**TITLE**			
**Title**	**1**	Identify the report as a systematic review.	**Yes**
BACKGROUND			
**Objectives**	**2**	Provide an explicit statement of the main objective(s) or question(s) the review addresses.	**Yes**
METHODS			
**Eligibility criteria**	**3**	Specify the inclusion and exclusion criteria for the review.	**Yes**
**Information sources**	**4**	Specify the information sources (e.g. databases, registers) used to identify studies and the date when each was last searched.	**Yes**
**Risk of bias**	**5**	Specify the methods used to assess risk of bias in the included studies.	**No**
**Synthesis of results**	**6**	Specify the methods used to present and synthesise results.	**Yes**
RESULTS			
**Included studies**	**7**	Give the total number of included studies and participants and summarise relevant characteristics of studies.	**Yes**
**Synthesis of results**	**8**	Present results for main outcomes, preferably indicating the number of included studies and participants for each. If meta-analysis was done, report the summary estimate and confidence/credible interval. If comparing groups, indicate the direction of the effect (i.e. which group is favoured).	**Yes**
DISCUSSION			
**Limitations of evidence**	**9**	Provide a brief summary of the limitations of the evidence included in the review (e.g. study risk of bias, inconsistency and imprecision).	**No**
**Interpretation**	**10**	Provide a general interpretation of the results and important implications.	**Yes**
OTHER			
**Funding**	**11**	Specify the primary source of funding for the review.	**No**
**Registration**	**12**	Provide the register name and registration number.	**No**

Table S2: Preferred Reporting Items for Systematic Reviews and Meta-Analysis (PRISMA) Checklist for the Manuscript

Section and Topic	Item #	Checklist item	Location where item is reported
**TITLE**			
**Title**	**1**	Identify the report as a systematic review.	**Page 1**
**ABSTRACT**			
**Abstract**	**2**	See the PRISMA 2020 for Abstracts checklist.	**Table S2**
**INTRODUCTION**			
**Rationale**	**3**	Describe the rationale for the review in the context of existing knowledge.	**Pages 2, 3, 4**
**Objectives**	**4**	Provide an explicit statement of the objective(s) or question(s) the review addresses.	**Page 4**
**METHODS**			
**Eligibility criteria**	**5**	Specify the inclusion and exclusion criteria for the review and how studies were grouped for the syntheses.	**Pages 4, 5**
**Information sources**	**6**	Specify all databases, registers, websites, organisations, reference lists and other sources searched or consulted to identify studies. Specify the date when each source was last searched or consulted.	**Page 4**
**Search strategy**	**7**	Present the full search strategies for all databases, registers and websites, including any filters and limits used.	**Page 4 and Table S3**
**Selection process**	**8**	Specify the methods used to decide whether a study met the inclusion criteria of the review, including how many reviewers screened each record and each report retrieved, whether they worked independently, and if applicable, details of automation tools used in the process.	**Pages 4, 5**
**Data collection process**	**9**	Specify the methods used to collect data from reports, including how many reviewers collected data from each report, whether they worked independently, any processes for obtaining or confirming data from study investigators, and if applicable, details of automation tools used in the process.	**Pages 4, 5**
**Data items**	**10a**	List and define all outcomes for which data were sought. Specify whether all results that were compatible with each outcome domain in each study were sought (e.g. for all measures, time points, analyses), and if not, the methods used to decide which results to collect.	**Pages 4, 5**
	**10b**	List and define all other variables for which data were sought (e.g. participant and intervention characteristics, funding sources). Describe any assumptions made about any missing or unclear information.	**Pages 4, 5**
**Study risk of bias assessment**	**11**	Specify the methods used to assess risk of bias in the included studies, including details of the tool(s) used, how many reviewers assessed each study and whether they worked independently, and if applicable, details of automation tools used in the process.	**Page 5**

**Effect measures**	**12**	Specify for each outcome the effect measure(s) (e.g. risk ratio, mean difference) used in the synthesis or presentation of results.	**Pages 4, 5**
**Synthesis methods**	**13a**	Describe the processes used to decide which studies were eligible for each synthesis (e.g. tabulating the study intervention characteristics and comparing against the planned groups for each synthesis (item #5)).	**Pages 4, 5**
	**13b**	Describe any methods required to prepare the data for presentation or synthesis, such as handling of missing summary statistics, or data conversions.	**Pages 4, 5**
	**13c**	Describe any methods used to tabulate or visually display results of individual studies and syntheses.	**Pages 4, 5**
	**13d**	Describe any methods used to synthesize results and provide a rationale for the choice(s). If meta-analysis was performed, describe the model(s), method(s) to identify the presence and extent of statistical heterogeneity, and software package(s) used.	**Pages 4, 5**
	**13e**	Describe any methods used to explore possible causes of heterogeneity among study results (e.g. subgroup analysis, meta-regression).	**Pages 4, 5**
	**13f**	Describe any sensitivity analyses conducted to assess robustness of the synthesized results.	**Pages 4, 5**
**Reporting bias assessment**	**14**	Describe any methods used to assess risk of bias due to missing results in a synthesis (arising from reporting biases).	**Pages 4, 5**
**Certainty assessment**	**15**	Describe any methods used to assess certainty (or confidence) in the body of evidence for an outcome.	**Not applicable**
**RESULTS**			
**Study selection**	**16a**	Describe the results of the search and selection process, from the number of records identified in the search to the number of studies included in the review, ideally using a flow diagram.	**Pages 5, 7, Figure 1, Table 1, Table S4**
	**16b**	Cite studies that might appear to meet the inclusion criteria, but which were excluded, and explain why they were excluded.	**Page 5, Figure 1**
**Study characteristics**	**17**	Cite each included study and present its characteristics.	**Pages 5, 7, Figure 2, Table 1, Table S4**
**Risk of bias in studies**	**18**	Present assessments of risk of bias for each included study.	**Page 7, Table S5**
**Results of individual studies**	**19**	For all outcomes, present, for each study: (a) summary statistics for each group (where appropriate) and (b) an effect estimate and its precision (e.g. confidence/credible interval), ideally using structured tables or plots.	**Page 5, 7, Figure 2, Figure 3**
**Results of syntheses**	**20a**	For each synthesis, briefly summarise the characteristics and risk of bias among contributing studies.	**Page 7, Table S5**

	**20b**	Present results of all statistical syntheses conducted. If meta-analysis was done, present for each the summary estimate and its precision (e.g. confidence/credible interval) and measures of statistical heterogeneity. If comparing groups, describe the direction of the effect.	**Pages 5, 7**
	**20c**	Present results of all investigations of possible causes of heterogeneity among study results.	**Page 7**
	**20d**	Present results of all sensitivity analyses conducted to assess the robustness of the synthesized results.	**Page 7**
**Reporting biases**	**21**	Present assessments of risk of bias due to missing results (arising from reporting biases) for each synthesis assessed.	**Page 7, Figure 3**
**Certainty of evidence**	**22**	Present assessments of certainty (or confidence) in the body of evidence for each outcome assessed.	**NA**
**DISCUSSION**			
**Discussion**	**23a**	Provide a general interpretation of the results in the context of other evidence.	**Pages 8, 9**
	**23b**	Discuss any limitations of the evidence included in the review.	**Pages 10, 11**
	**23c**	Discuss any limitations of the review processes used.	**Pages 10, 11**
	**23d**	Discuss implications of the results for practice, policy, and future research.	**Pages 8, 9, 10, 11**
**OTHER INFORMATION**			
**Registration and protocol**	**24a**	Provide registration information for the review, including register name and registration number, or state that the review was not registered.	**Page 4**
	**24b**	Indicate where the review protocol can be accessed, or state that a protocol was not prepared.	**Page 4**
	**24c**	Describe and explain any amendments to information provided at registration or in the protocol.	**Page 4**
**Support**	**25**	Describe sources of financial or non-financial support for the review, and the role of the funders or sponsors in the review.	**Page 11**
**Competing interests**	**26**	Declare any competing interests of review authors.	**Page 11**
**Availability of data, code and other materials**	**27**	Report which of the following are publicly available and where they can be found: template data collection forms; data extracted from included studies; data used for all analyses; analytic code; any other materials used in the review.	**Page 4, Table S3**

Table S3: Full Search Strategy

MEDLINE(R) ALL (Ovid) 1946 to May 07, 2025

#	Searches	Results
1	exp Vulvar Neoplasms/	9152
2	((Paget* or vulv* or (Lower adj2 Female adj2 Genital)) adj4 (cancer* or oncolog* or malign* or carcin* or tumor* or tumour* or neoplas* or sarcoma* or adenocarcinoma* or adenosarcoma*)).tw,kf.	9839
3	1 or 2	13265
4	exp Receptor, ErbB-2/	31945
5	exp Genes, erbB-2/	3086
6	((human adj2 epidermal adj2 growth adj2 factor*) or erbB2* or erbb-2* or HER2* or HER-2*).ab,kf,ti.	65265
7	4 or 5 or 6	69784
8	3 and 7	129

Embase Classic+Embase 1947 to 2025 May 09

#	Searches	Results
1	exp vulva tumor/	17771
2	((Paget* or vulv* or (Lower adj2 Female adj2 Genital)) adj4 (cancer* or oncolog* or malign* or carcin* or tumor* or tumour* or neoplas* or sarcoma* or adenocarcinoma* or adenosarcoma*)).tw,kf.	15457
3	1 or 2	23407
4	exp epidermal growth factor receptor 2/	86144
5	(gene* adj4 (erbB?2* or her2* or her?2*)).ab,kf,ti.	6910
6	((human adj2 epidermal adj2 growth adj2 factor*) or erbB2* or erbb-2* or HER2* or HER-2*).ab,kf,ti.	119792
7	4 or 5 or 6	143717
8	3 and 7	411

Cochrane Library 1946 to May 12, 2025

ID	Search	Hits
#1	MeSH descriptor: [Vulvar Neoplasms] explode all trees	164
#2	((Paget* or vulv* or (Lower adj2 Female NEAR/2 Genital)) NEAR/4 (cancer* or oncolog* or malign* or carcin* or tumor* or tumour* or neoplas* or sarcoma* or adenocarcinoma* or adenosarcoma*)):ab,ti	2879
#3	#1 or #2	2940

#4	MeSH descriptor: [Receptor, ErbB-2] explode all trees	1702
#5	MeSH descriptor: [Genes, erbB-2] explode all trees	73
#6	((human NEAR/3 epidermal NEAR/3 growth NEAR/3 factor* NEAR/3 receptor*) or HER2 or HER-2):ab,ti	9495
#7	(gene* NEAR/4 (erbB2 or erbb?2 or her2* or her?2*)):ab,ti	294
#8	#4 or #5 or #6 or #7	9708
#9	#3 and #8	76

Table S4: A table of studies that reported on HER2 expression among patients with predominantly vulvar squamous cell carcinoma with associated clinicopathological characteristics.

Study by Author	country	Year	N	ASCO/CAP guidelines	Age	p53 total (N)	p53 positive (N)	HPV total (N)	HPV positive (N)	LN+ (N)	LN- (N)	Data on Survival
Berchuck et al. [44]	USA	1990	34	No								Heavy HER2 staining was noted in one patient with distant metastases.
Gordinier et al. [45]	USA	1997	39	No		39	25			13	26	There was association of HER2 expression with nodal metastasis.
Hantschmann et al. [46]	Germany	2005	75	No						21	25	HER2 expression did not impact survival.
Dedes et al. [43]	Switzerland	2011	59	ASCO								
Choschzick et al. [38]	Germany	2013	154	ASCO	55 years (range: 43–62)					1	2	
Palisoul et al. [40]	USA	2017	143	ASCO		66	22					HER2 expression was more common in adenocarcinoma than squamous cell carcinoma of the vulva.
Baiocchi et al. [39]	Brazil	2021	117	ASCO				27	13	80	45	ERBB family expression was not associated with worse survival.
Garganese et al. [42]	Italy	2021	101	ASCO	78 (range 48–96)	101	75	101	14	48	53	
Pauly et al. [41]	Germany	2025	47	ASCO	69					10	37	

Abbreviations: N: number of cases, ASCO/CAP: American Society of Clinical Oncology/College of American Pathologists, HER2: human epidermal growth factor receptor 2, p53: protein 53, HPV: human papilloma virus, LN+: number of patients with lymph node positive status, LN-: number of patients with lymph node negative status.

Table S5: Newcastle-Ottawa Scale assessment of cohort studies

Study	Selection	Comparability	Outcome	Score
Berchuck et al. [44]	3	2	1	6
Gordinier et al. [45]	3	1	3	7
Hantschmann et al. [46]	3	1	2	6
Dedes et al. [43]				Unable to score
Choschzick et al. [38]	3	1	2	6
Palisoul et al. [40]	3	2	3	8
Baiocchi et al. [39]	3	2	3	8
Garganese et al. [42]	4	2	3	9
Pauly et al. [41]				Unable to score
